# 2-Eth­oxy-4-{[(2-nitro­phen­yl)hydrazono]meth­yl}phenol

**DOI:** 10.1107/S160053680903699X

**Published:** 2009-09-30

**Authors:** Zhi-Gang Yin, Heng-Yu Qian, Chun-Xia Zhang, Xue-Wen Zhu

**Affiliations:** a Key Laboratory of Surface and Interface Science of Henan, School of Materials and Chemical Engineering, Zhengzhou University of Light Industry, Zhengzhou 450002, People’s Republic of China

## Abstract

The title compound, C_15_H_15_N_3_O_4_, a Schiff base, was obtained from a condensation reaction of 3-eth­oxy-4-hydroxy­benzaldehyde and 2-nitro­phenyl­hydrazine. The mol­ecule is approximately planar, the largest deviation from the mean plane being 0.1449 (16) Å. An intramolecular N—H⋯O inter­action is also present. In the crystal, inter­molecular O—H⋯O hydrogen bonds link the mol­ecules, forming chain parallel to the *b* axis.

## Related literature

For the role played by Schiff bases in the development of various proteins and enzymes, see: Kahwa *et al.* (1986 ▶); Santos *et al.* (2001 ▶).
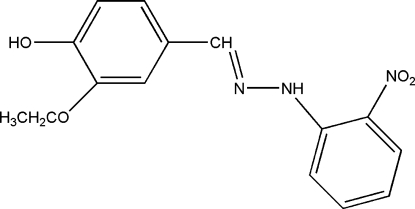


## Experimental

### 

#### Crystal data

C_15_H_15_N_3_O_4_
*M* *_r_* = 301.30
Monoclinic, 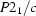

*a* = 14.586 (3) Å
*b* = 5.002 (1) Å
*c* = 19.894 (4) Å
β = 102.40 (3)°
*V* = 1417.6 (5) Å^3^
*Z* = 4
Mo *K*α radiation
μ = 0.11 mm^−1^
*T* = 296 K
0.20 × 0.18 × 0.17 mm

#### Data collection

Bruker SMART CCD area-detector diffractometer
Absorption correction: multi-scan (*SADABS*; Bruker, 1998 ▶) *T*_min_ = 0.979, *T*_max_ = 0.982
5585 measured reflections
2762 independent reflections
1264 reflections with *I* > 2σ(*I*)
*R*_int_ = 0.040

#### Refinement

*R*[*F* ^2^ > 2σ(*F* ^2^)] = 0.037
*wR*(*F* ^2^) = 0.071
*S* = 0.72
2762 reflections
201 parameters
H-atom parameters constrained
Δρ_max_ = 0.13 e Å^−3^
Δρ_min_ = −0.20 e Å^−3^

### 

Data collection: *SMART* (Bruker, 1998 ▶); cell refinement: *SAINT* (Bruker, 1998 ▶); data reduction: *SAINT*; program(s) used to solve structure: *SHELXS97* (Sheldrick, 2008 ▶); program(s) used to refine structure: *SHELXL97* (Sheldrick, 2008 ▶); molecular graphics: *ORTEPIII* (Burnett & Johnson, 1996 ▶), *ORTEP-3 for Windows* (Farrugia, 1997 ▶) and *PLATON* (Spek, 2009 ▶); software used to prepare material for publication: *SHELXL97*.

## Supplementary Material

Crystal structure: contains datablocks global, I. DOI: 10.1107/S160053680903699X/dn2485sup1.cif

Structure factors: contains datablocks I. DOI: 10.1107/S160053680903699X/dn2485Isup2.hkl

Additional supplementary materials:  crystallographic information; 3D view; checkCIF report

## Figures and Tables

**Table 1:** Hydrogen-bond geometry (Å, °)

*D*—H⋯*A*	*D*—H	H⋯*A*	*D*⋯*A*	*D*—H⋯*A*
N2—H2⋯O1	0.86	1.99	2.610 (2)	128
O4—H11⋯O4^i^	0.82	2.21	2.9842 (16)	159

Symmetry code: (i) 
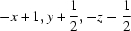
.

**Fig. 1.**
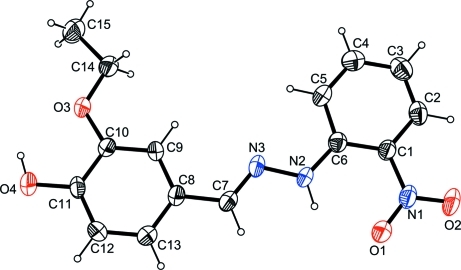
Molecular view of of (I) with the atom labeling scheme. Displacement ellipsoids are drawn at the 30% probability level. H atoms are represented as small spheres of arbitrary radii.

**Fig. 2.**
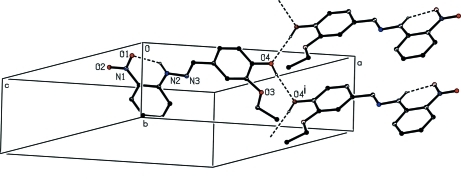
Partial packing view of (I) showing the formation of a chain through O-H···O hydrogen bonds. H atoms are represented as small spheres of arbitrary radii and H bonds are shown as dashed line. H atoms not involved in hydrogen bonding have been omitted for clarity.[Symmetry codes: (i) -x+1, y+1/2, -z-1/2]

## supplementary crystallographic information

### Comment

The chemistry of Schiff base has attracted a great deal of interest in recent years. These compounds play an important role in the development of various proteins and enzymes (Kahwa *et al.*, 1986; Santos *et al.*, 2001). As part of our interest  in the study of the coordination chemistry of Schiff bases, we synthesized the title compound and determined its crystal structure.

The whole molecule is roughly planar with the largest deviation from the mean plane being -0.1449 (16)Å at C15 (Fig. 1).

Intermolecular O—H···O hydrogen bonds link the molecule to form chain parallel to the b axis (Table 1, Fig. 2).

### Experimental

2-Nitrophenylhydrazine (1 mmol, 0.153 g) was dissolved in anhydrous ethanol (15 ml), The mixture was stirred for several minitutes at 351k, 3-Ethoxy-4-hydroxybenzaldehyde (1 mmol, 0.166 g) in ethanol (8 mm l) was added dropwise and the mixture was stirred at refluxing temperature for 2 h. The product was isolated and recrystallized from methanol, red single crystals of (I) was obtained after 3 d.

### Refinement

All H atoms were positioned geometrically and refined as riding with C—H=0.93 (aromatic), 0.97(methylene), 0.96 Å(methyl) and N—H=0.86 Å, with *U*_iso_(H)=1.2*U*_eq_(CH, CH2 or NH) and *U*_iso_(H)=1.5*U*_eq_(C).

### Crystal data

**Table:**

C_15_H_15_N_3_O_4_	*F*(000) = 632
*M**_r_* = 301.30	*D*_x_ = 1.412 Mg m^−^^3^
Monoclinic, *P*2_1_/*c*	Mo *K*α radiation, λ = 0.71073 Å
Hall symbol: -P 2ybc	Cell parameters from 1993 reflections
*a* = 14.586 (3) Å	θ = 3.1–28.2°
*b* = 5.002 (1) Å	µ = 0.11 mm^−^^1^
*c* = 19.894 (4) Å	*T* = 296 K
β = 102.40 (3)°	Block, red
*V* = 1417.6 (5) Å^3^	0.20 × 0.18 × 0.17 mm
*Z* = 4	

### Data collection

**Table:**

Bruker SMART CCD area-detector diffractometer	2762 independent reflections
Radiation source: fine-focus sealed tube	1264 reflections with *I* > 2σ(*I*)
graphite	*R*_int_ = 0.040
ω scans	θ_max_ = 26.0°, θ_min_ = 3.2°
Absorption correction: multi-scan (*SADABS*; Bruker, 1998)	*h* = −17→16
*T*_min_ = 0.979, *T*_max_ = 0.982	*k* = −6→5
5585 measured reflections	*l* = −17→24

### Refinement

**Table:**

Refinement on *F*^2^	Primary atom site location: structure-invariant direct methods
Least-squares matrix: full	Secondary atom site location: difference Fourier map
*R*[*F*^2^ > 2σ(*F*^2^)] = 0.037	Hydrogen site location: inferred from neighbouring sites
*wR*(*F*^2^) = 0.071	H-atom parameters constrained
*S* = 0.72	*w* = 1/[σ^2^(*F*_o_^2^) + (0.0305*P*)^2^] where *P* = (*F*_o_^2^ + 2*F*_c_^2^)/3
2762 reflections	(Δ/σ)_max_ < 0.001
201 parameters	Δρ_max_ = 0.13 e Å^−^^3^
0 restraints	Δρ_min_ = −0.20 e Å^−^^3^

### Special details

**Table:**

Geometry. All esds (except the esd in the dihedral angle between two l.s. planes) are estimated using the full covariance matrix. The cell esds are taken into account individually in the estimation of esds in distances, angles and torsion angles; correlations between esds in cell parameters are only used when they are defined by crystal symmetry. An approximate (isotropic) treatment of cell esds is used for estimating esds involving l.s. planes.
Refinement. Refinement of *F*^2^ against ALL reflections. The weighted *R*-factor *wR* and goodness of fit *S* are based on *F*^2^, conventional *R*-factors *R* are based on *F*, with *F* set to zero for negative *F*^2^. The threshold expression of *F*^2^ > σ(*F*^2^) is used only for calculating *R*-factors(gt) *etc*. and is not relevant to the choice of reflections for refinement. *R*-factors based on *F*^2^ are statistically about twice as large as those based on *F*, and *R*- factors based on ALL data will be even larger.

### Fractional atomic coordinates and isotropic or equivalent isotropic displacement parameters (Å^2^)

**Table:**

	*x*	*y*	*z*	*U*_iso_*/*U*_eq_	
C1	0.06946 (14)	0.4950 (3)	0.12464 (9)	0.0311 (5)	
C2	0.07330 (15)	0.6553 (4)	0.18267 (10)	0.0385 (5)	
H2A	0.0301	0.6290	0.2103	0.046*	
C3	0.13985 (16)	0.8501 (4)	0.19918 (10)	0.0435 (6)	
H3	0.1428	0.9562	0.2380	0.052*	
C4	0.20308 (15)	0.8864 (4)	0.15677 (10)	0.0431 (6)	
H4	0.2483	1.0197	0.1674	0.052*	
C5	0.20040 (15)	0.7316 (4)	0.10010 (10)	0.0404 (6)	
H5	0.2439	0.7620	0.0729	0.049*	
C6	0.13368 (14)	0.5272 (3)	0.08161 (10)	0.0309 (5)	
C7	0.19806 (14)	0.2628 (4)	−0.06428 (9)	0.0350 (5)	
H7	0.1510	0.1341	−0.0737	0.042*	
C8	0.26434 (14)	0.2814 (3)	−0.10903 (9)	0.0320 (5)	
C9	0.33800 (14)	0.4669 (3)	−0.09704 (9)	0.0320 (5)	
H9	0.3455	0.5822	−0.0596	0.038*	
C10	0.39910 (14)	0.4781 (3)	−0.14074 (10)	0.0315 (5)	
C11	0.38746 (14)	0.3060 (4)	−0.19732 (9)	0.0310 (5)	
C12	0.31578 (15)	0.1243 (4)	−0.20896 (10)	0.0369 (5)	
H12	0.3082	0.0102	−0.2467	0.044*	
C13	0.25466 (15)	0.1098 (4)	−0.16481 (10)	0.0375 (5)	
H13	0.2066	−0.0161	−0.1726	0.045*	
C14	0.49775 (15)	0.8147 (4)	−0.07617 (10)	0.0397 (5)	
H14A	0.5067	0.7107	−0.0341	0.048*	
H14B	0.4476	0.9422	−0.0764	0.048*	
C15	0.58674 (16)	0.9580 (4)	−0.08049 (11)	0.0557 (7)	
H15A	0.6358	0.8298	−0.0802	0.084*	
H15B	0.6044	1.0757	−0.0417	0.084*	
H15C	0.5769	1.0602	−0.1223	0.084*	
N1	−0.00228 (12)	0.2929 (3)	0.11143 (9)	0.0380 (4)	
N2	0.13414 (11)	0.3742 (3)	0.02520 (8)	0.0365 (4)	
H2	0.0924	0.2519	0.0131	0.044*	
N3	0.20184 (11)	0.4161 (3)	−0.01266 (8)	0.0345 (4)	
O1	−0.00973 (10)	0.1463 (3)	0.06016 (7)	0.0466 (4)	
O2	−0.05468 (11)	0.2664 (3)	0.15192 (7)	0.0572 (5)	
O3	0.47481 (10)	0.6437 (2)	−0.13471 (7)	0.0433 (4)	
O4	0.44797 (11)	0.3159 (2)	−0.24127 (7)	0.0462 (4)	
H11	0.4789	0.4540	−0.2346	0.069*	

### Atomic displacement parameters (Å^2^)

**Table:**

	*U*^11^	*U*^22^	*U*^33^	*U*^12^	*U*^13^	*U*^23^
C1	0.0301 (13)	0.0326 (11)	0.0327 (12)	−0.0013 (10)	0.0117 (10)	0.0023 (9)
C2	0.0433 (15)	0.0418 (12)	0.0346 (12)	0.0052 (12)	0.0179 (11)	0.0040 (10)
C3	0.0542 (16)	0.0416 (13)	0.0356 (13)	0.0044 (12)	0.0119 (12)	−0.0022 (10)
C4	0.0453 (16)	0.0383 (12)	0.0458 (14)	−0.0061 (11)	0.0099 (12)	−0.0029 (11)
C5	0.0400 (15)	0.0406 (12)	0.0456 (14)	−0.0100 (11)	0.0201 (12)	0.0002 (10)
C6	0.0321 (13)	0.0320 (11)	0.0296 (12)	0.0000 (10)	0.0090 (10)	0.0023 (9)
C7	0.0316 (13)	0.0389 (11)	0.0371 (12)	−0.0088 (10)	0.0131 (11)	−0.0003 (10)
C8	0.0311 (13)	0.0342 (11)	0.0343 (12)	−0.0021 (10)	0.0151 (10)	−0.0006 (10)
C9	0.0372 (14)	0.0326 (11)	0.0306 (12)	−0.0039 (10)	0.0173 (11)	−0.0038 (9)
C10	0.0332 (13)	0.0286 (11)	0.0363 (12)	−0.0039 (10)	0.0156 (10)	−0.0011 (9)
C11	0.0349 (13)	0.0348 (12)	0.0276 (11)	0.0034 (10)	0.0163 (10)	0.0018 (9)
C12	0.0450 (14)	0.0373 (12)	0.0305 (12)	−0.0057 (11)	0.0128 (11)	−0.0082 (9)
C13	0.0382 (14)	0.0388 (12)	0.0378 (13)	−0.0110 (10)	0.0132 (11)	−0.0044 (10)
C14	0.0448 (14)	0.0419 (11)	0.0345 (12)	−0.0099 (11)	0.0134 (11)	−0.0002 (10)
C15	0.0494 (16)	0.0683 (15)	0.0527 (15)	−0.0233 (13)	0.0186 (13)	−0.0084 (12)
N1	0.0358 (12)	0.0444 (10)	0.0388 (10)	−0.0033 (9)	0.0187 (9)	0.0033 (9)
N2	0.0353 (11)	0.0403 (9)	0.0398 (11)	−0.0130 (8)	0.0213 (9)	−0.0045 (8)
N3	0.0310 (11)	0.0416 (10)	0.0355 (10)	−0.0039 (8)	0.0173 (9)	0.0014 (8)
O1	0.0463 (10)	0.0544 (9)	0.0444 (10)	−0.0174 (8)	0.0215 (8)	−0.0101 (7)
O2	0.0517 (11)	0.0749 (10)	0.0563 (10)	−0.0173 (9)	0.0363 (9)	−0.0046 (8)
O3	0.0441 (9)	0.0484 (8)	0.0459 (9)	−0.0189 (8)	0.0284 (8)	−0.0145 (7)
O4	0.0547 (11)	0.0470 (9)	0.0472 (9)	−0.0078 (8)	0.0339 (8)	−0.0069 (7)

### Geometric parameters (Å, °)

**Table:**

C1—C2	1.397 (2)	C10—O3	1.365 (2)
C1—C6	1.407 (3)	C10—C11	1.398 (2)
C1—N1	1.438 (2)	C11—C12	1.367 (2)
C2—C3	1.365 (3)	C11—O4	1.370 (2)
C2—H2A	0.9300	C12—C13	1.381 (3)
C3—C4	1.389 (3)	C12—H12	0.9300
C3—H3	0.9300	C13—H13	0.9300
C4—C5	1.361 (2)	C14—O3	1.426 (2)
C4—H4	0.9300	C14—C15	1.501 (3)
C5—C6	1.405 (2)	C14—H14A	0.9700
C5—H5	0.9300	C14—H14B	0.9700
C6—N2	1.359 (2)	C15—H15A	0.9600
C7—N3	1.273 (2)	C15—H15B	0.9600
C7—C8	1.451 (3)	C15—H15C	0.9600
C7—H7	0.9300	N1—O2	1.2308 (19)
C8—C13	1.386 (2)	N1—O1	1.2417 (18)
C8—C9	1.401 (2)	N2—N3	1.380 (2)
C9—C10	1.373 (3)	N2—H2	0.8600
C9—H9	0.9300	O4—H11	0.8200
			
C2—C1—C6	121.55 (18)	C12—C11—O4	119.34 (17)
C2—C1—N1	116.94 (18)	C12—C11—C10	120.12 (17)
C6—C1—N1	121.50 (17)	O4—C11—C10	120.54 (18)
C3—C2—C1	120.59 (19)	C11—C12—C13	120.11 (18)
C3—C2—H2A	119.7	C11—C12—H12	119.9
C1—C2—H2A	119.7	C13—C12—H12	119.9
C2—C3—C4	118.55 (19)	C12—C13—C8	120.53 (19)
C2—C3—H3	120.7	C12—C13—H13	119.7
C4—C3—H3	120.7	C8—C13—H13	119.7
C5—C4—C3	121.6 (2)	O3—C14—C15	107.09 (16)
C5—C4—H4	119.2	O3—C14—H14A	110.3
C3—C4—H4	119.2	C15—C14—H14A	110.3
C4—C5—C6	121.73 (19)	O3—C14—H14B	110.3
C4—C5—H5	119.1	C15—C14—H14B	110.3
C6—C5—H5	119.1	H14A—C14—H14B	108.6
N2—C6—C5	119.98 (17)	C14—C15—H15A	109.5
N2—C6—C1	124.03 (18)	C14—C15—H15B	109.5
C5—C6—C1	115.98 (17)	H15A—C15—H15B	109.5
N3—C7—C8	122.48 (18)	C14—C15—H15C	109.5
N3—C7—H7	118.8	H15A—C15—H15C	109.5
C8—C7—H7	118.8	H15B—C15—H15C	109.5
C13—C8—C9	119.29 (18)	O2—N1—O1	121.13 (17)
C13—C8—C7	118.99 (18)	O2—N1—C1	119.06 (16)
C9—C8—C7	121.72 (17)	O1—N1—C1	119.80 (16)
C10—C9—C8	119.83 (17)	C6—N2—N3	119.74 (16)
C10—C9—H9	120.1	C6—N2—H2	120.1
C8—C9—H9	120.1	N3—N2—H2	120.1
O3—C10—C9	126.29 (18)	C7—N3—N2	115.88 (16)
O3—C10—C11	113.60 (16)	C10—O3—C14	118.65 (14)
C9—C10—C11	120.11 (19)	C11—O4—H11	109.5

### Hydrogen-bond geometry (Å, °)

**Table:**

*D*—H···*A*	*D*—H	H···*A*	*D*···*A*	*D*—H···*A*
N2—H2···O1	0.86	1.99	2.610 (2)	128
O4—H11···O4^i^	0.82	2.21	2.9842 (16)	159

Symmetry codes: (i) −*x*+1, *y*+1/2, −*z*−1/2.
